# Sleep and Core Body Temperature Alterations Induced by Space Radiation in Rats

**DOI:** 10.3390/life13041002

**Published:** 2023-04-13

**Authors:** Larry D. Sanford, Austin M. Adkins, Alea F. Boden, Justin D. Gotthold, Ryan D. Harris, Dorela Shuboni-Mulligan, Laurie L. Wellman, Richard A. Britten

**Affiliations:** 1 Sleep Research Laboratory, Center for Integrative Neuroscience and Inflammatory Diseases, Pathology and Anatomy, Eastern Virginia Medical School, Norfolk, VA 23507, USA; 2 Center for Integrative Neuroscience and Inflammatory Diseases, Radiation Oncology, Eastern Virginia Medical School, Norfolk, VA 23507, USA

**Keywords:** core body temperature, EEG, sleep, space radiation

## Abstract

Sleep problems in astronauts can arise from mission demands and stress and can impact both their health and ability to accomplish mission objectives. In addition to mission-related physical and psychological stressors, the long durations of the proposed Mars missions will expose astronauts to space radiation (SR), which has a significant impact on the brain and may also alter sleep and physiological functions. Therefore, in this study, we assessed sleep, EEG spectra, activity, and core body temperature (CBT) in rats exposed to SR and compared them to age-matched nonirradiated rats. Male outbred Wistar rats (8–9 months old at the time of the study) received SR (15 cGy GCRsim, *n* = 15) or served as age- and time-matched controls (CTRL, *n* = 15) without irradiation. At least 90 days after SR and 3 weeks prior to recording, all rats were implanted with telemetry transmitters for recording EEG, activity, and CBT. Sleep, EEG spectra (delta, 0.5–4 Hz; theta, 4–8 Hz; alpha, 8–12 Hz; sigma, 12–16 Hz; beta, 16–24 Hz), activity, and CBT were examined during light and dark periods and during waking and sleeping states. When compared to the CTRLs, SR produced significant reductions in the amounts of dark period total sleep time, total nonrapid eye movement sleep (NREM), and total rapid eye movement sleep (REM), with significant decreases in light and dark period NREM deltas and dark period REM thetas as well as increases in alpha and sigma in NREM and REM during either light or dark periods. The SR animals showed modest increases in some measures of activity. CBT was significantly reduced during waking and sleeping in the light period. These data demonstrate that SR alone can produce alterations to sleep and temperature control that could have consequences for astronauts and their ability to meet mission demands.

## 1. Introduction

Humans evolved in the unique gravity, lighting, atmospheric, and environmental conditions of Earth. These conditions provided the scaffold for the development of our physiology, and the challenges of adapting to our environment helped guide the development of our cognition, emotional abilities, and social structures. However, spaceflight and ambitions for interplanetary exploration have taken humans away from the relatively benign and familiar comfort of Earth and placed our astronaut emissaries in a harsh and unforgiving environment where support for basic physiological needs must be provided by technology and where social and psychological support is limited. Understanding the challenges that the space environment places on human physiology and psychology will provide the information needed to best support successful human space missions.

Life on Earth is marked by periodic alterations in physiological functions, including states of arousal and sleep. In space and on Mars, many of the environmental cues that control the timing of these functions that can impact sleep will be lost or changed. Good quality sleep in sufficient quantity is a core need for human health and performance. The dysregulation and disturbance of sleep are associated with an increase in chronic inflammatory diseases [1,2], as well as a large range of physical diseases and neuropsychiatric disorders. Sleep has also long been implicated in learning, memory and cognitive performance, including facilitating the underlying neural activity and communication between brain regions [3,4,5,6]. Thus, maintaining sufficient good quality sleep will be critical for ensuring astronaut health and performance and mission success.

Studies have found that astronauts in space sleep less than they normally do on Earth due to multiple factors (e.g., noise, physical discomfort, altered light-dark cycles, varying work shifts, hypoxia, hypercapnia, and psychological factors) [7,8,9,10]. Some physical discomfort may be associated with microgravity, which requires that astronauts sleep in tethered bags and can cause back pain in some astronauts [11]. Microgravity also has documented effects on the microbiome and gut mucosa, as well as the skeletal, sensorimotor, and ocular systems and the brain (e.g., [12,13,14,15,16,17,18,19]), which may also impact sleep. Additionally, data from ground-based analogs for team cohesion and astronaut psychological status suggest that some astronauts will have problems with sleep, interpersonal interactions, stress, and issues related to isolation (Mars 500, Antarctic confinement studies). Social isolation is a significant concern for astronauts on interplanetary missions who will spend prolonged periods confined within a spacecraft with limited social interactions and separation from their families and social support networks. Work in both humans and animals has demonstrated the deleterious effects of social isolation on multiple systems [20].

In addition to the physical and psychological stressors demonstrated in spaceflight and simulated missions, astronauts on the planned mission to Mars also will be unavoidably exposed to space radiation (SR). The deep SR spectrum (galactic cosmic radiation (GCR)) is composed of highly energetic protons, helium nuclei, and high-mass (Z ≤ 28) charged (HZE) ions, with ~50% of the dose accruing from high-energy protons [21]. Astronauts are predicted to be exposed to around 13 cGy per year [21]. In ground-based rodent studies, SR can alter neurotransmission in multiple brain regions (reviewed in [22]) with the potential for injurious effects across multiple functional systems. Rodent studies have demonstrated SR-induced impairments in cognition (reviewed in [22,23,24,25,26]), psycho-social behavior [27,28,29,30,31,32], and sensorimotor function, including gross [33,34,35,36] and fine motor [37,38] skills. The potential effects of SR on sleep have been minimally studied [22], but the role of sleep in health and performance across multiple domains suggests that it will be imperative to understand how SR impacts sleep before humans are sent into deep space. Therefore, in this study, we assessed the effects of 15 cGy GCRsim on sleep in rats by examining overall sleep amount and EEG spectra across arousal and sleep states. We also determined the effects of SR on core body temperature (CBT), which is closely associated with sleep [39,40,41], can increase in astronauts during extended time in space [42], and can be elevated with stress [43].

## 2. Materials and Methods

### 2.1. Subjects

Retired male breeder (outbred, Wistar) rats were obtained from Hilltop Lab Animals, Inc., Scottsdale, PA, USA. At EVMS, the rats were single-housed in rooms kept on a 12:12 light:dark cycle, with ambient temperature maintained at 24.5 ± 0.5 °C. Teklad 2014 chow and water were available ad libitum. All studies were conducted in accordance with the National Research Council’s “Guide for the Care and Use of Laboratory Animals (8th Edition)” at the animal care facilities of Eastern Virginia Medical School (EVMS) and Brookhaven National Laboratory (BNL), both of which are accredited by the Association for Assessment and Accreditation of Laboratory Animal Care, International. Relevant procedures were approved by the Institutional Animal Care and Use Committees (IACUC) of both EVMS and BNL, depending on where they were conducted.

### 2.2. Irradiation Procedure

Rats were shipped to BNL, where they were single-housed and given ad libitum access to Teklad 2014 chow and water. After at least one week of acclimatization, the rats were randomly assigned to receive whole-body irradiation with 0 (sham) or 15 cGy GCRsim (SR) at the NASA Space Radiation Laboratory (NSRL). The rats were placed in a well-ventilated custom-made irradiation jig that was constructed of black polyacrylic plastic and exposed to the simplified 5-ion GCRSim at an overall dose rate of 0.5 cGy/min (~20 min exposure). Dose calibration was performed as previously described [44]. Sham rats were placed in an identical irradiation jig that remained in the preparation room while their counterparts were taken into the radiation vault at NSRL.

One week after irradiation, the SR (*n* = 15) and sham (*n* = 5) rats were transported back to EVMS. No significant differences in behavior or sleep parameters between the unshipped control (CTRL) and sham groups were observed (see Supplemental Table S1 and Figures S1 and S2). Therefore, the sham rats were incorporated into the respective CTRL groups for subsequent analyses (CTRL: *n* = 15 and SR: *n* = 15). The group size represents the size of one cohort that can be irradiated concurrently and matched to an identical number of nonirradiated animals. This number also provides > 0.8 power to determine differences in most of the sleep, EEG, and CBT parameters.

### 2.3. Surgery

At least 90 days post-irradiation (or equivalent time for CTRL animals), the rats were implanted intraperitoneally with telemetry transmitters (ETA F10, Data Sciences International; St. Paul, MN, USA) to record EEG activity, gross body activity, and CBT. Leads from the transmitter were led subcutaneously to the head, and the free ends were placed into holes drilled in the dorsal skull to allow for recording EEG. All surgeries were conducted under isoflurane (inhalant: 5% induction; 2–3% maintenance) anesthesia. Ibuprofen (30 mg/kg, oral) was continuously available in each animal’s drinking water for 24–48 h pre-operatively and for a minimum of 72 h post-operatively for pain relief. All animals received prophylactic procaine penicillin (22,000–100,000 IU/kg), gentamicin (5–8 mg/kg), and dexamethasone (0.5–2 mg/kg) subcutaneously on the day of surgery.

### 2.4. Sleep Recording and Scoring

Sleep was recorded as a component of a series of behavioral tests that began approximately 90 days post-irradiation and continued up to 160 days post-irradiation. Behavioral testing for anticipatory activity, adhesive removal, novel object recognition, and balance beam performance were conducted, followed by baseline sleep recording. At least two undisturbed days elapsed between the last behavioral test and sleep recording.

Baseline sleep recordings were obtained for 20 h periods across the groups. Recordings were visually scored using Data Sciences International Neuroscore^TM^ Rodent Sleep Module. Observers were blind to the conditions and scored the records in 10 s epochs to determine wakefulness, NREM, and REM. Wakefulness was scored based on the presence of low-voltage fast EEG and high activity levels and categorized as active waking (AW) or quiet waking (QW) based on the presence or absence of activity, i.e., during AW, the animals were active, and during QW, the animals were awake but inactive/resting. NREM was characterized by the presence of spindles interspersed with slow waves and no gross body movements. REM was scored continuously during the presence of low voltage, fast EEG, theta rhythm, and no activity. Data were separated into 8 h light periods and 12 h dark periods across the 20 h recording period. The following sleep parameters were examined in the data analyses: total NREM (min), total REM (min); total sleep time (TST: REM + NREM), REM% (REM/TST*100), and number and durations of NREM and REM episodes (defined as contiguous 10 s epochs of a given state). Total NREM and REM were also examined hourly across the recording period.

### 2.5. EEG Analyses

EEG signals were digitized at 256 Hz and spectrally analyzed by fast Fourier transformation (FFT) using the Neuroscore™ program. The relative power in five spectral bands (delta, 0.5–4 Hz; theta, 4–8 Hz; alpha, 8–12 Hz; sigma, 12–16 Hz; beta, 16–24 Hz) was examined during QW, NREM, and REM and were compared across treatment groups for each behavioral state during light and dark period recordings.

### 2.6. Activity Analyses

Activity was analyzed using both total activity counts, as recorded by the telemetry implants, and active time, calculated as the number of 10 s epochs in which activity was recorded. These data were analyzed across the 8 h light periods and 12 h dark periods and compared across treatment groups. Active time was also examined hourly across the recording period.

### 2.7. Temperature Analyses

CBT was evaluated for AW, QW, NREM, and REM for the 8 h light period and 12 h dark period and compared across treatment groups. Temperature was also examined hourly across the recording period.

### 2.8. Statistical Analyses

Sleep, EEG, activity, and temperature data were analyzed using one-way (across treatments) ANOVAs or *t*-tests for the light and dark periods using SigmaPlot analysis software. Hourly sleep, active time, and temperature data were analyzed using two-way mixed factors ANOVAs with repeated measures on time. Post-hoc comparison tests for significant ANOVAs were performed using the Holm-Sidak method.

## 3. Results

### 3.1. Sleep Amounts

**Figure 1:**
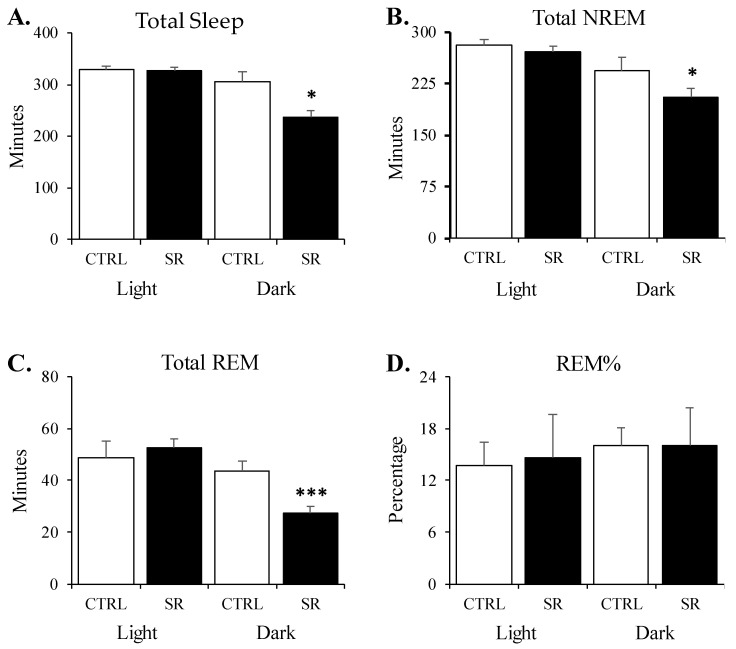
**Sleep amounts in control (CTRL) and irradiated (SR) rats during the 8 h light period and 12 h dark period.** (**A**) total time spent asleep; (**B**) total nonrapid eye movement (NREM) sleep; (**C**) total rapid eye movement (REM) sleep; (**D**) REM% (percentage of total sleep time). *, *p* < 0.05; ***, *p* < 0.001 for comparisons between CTRL (*n* = 15) and SR (*n* = 15). Data are presented as mean ± SEM.

**Figure 2:**
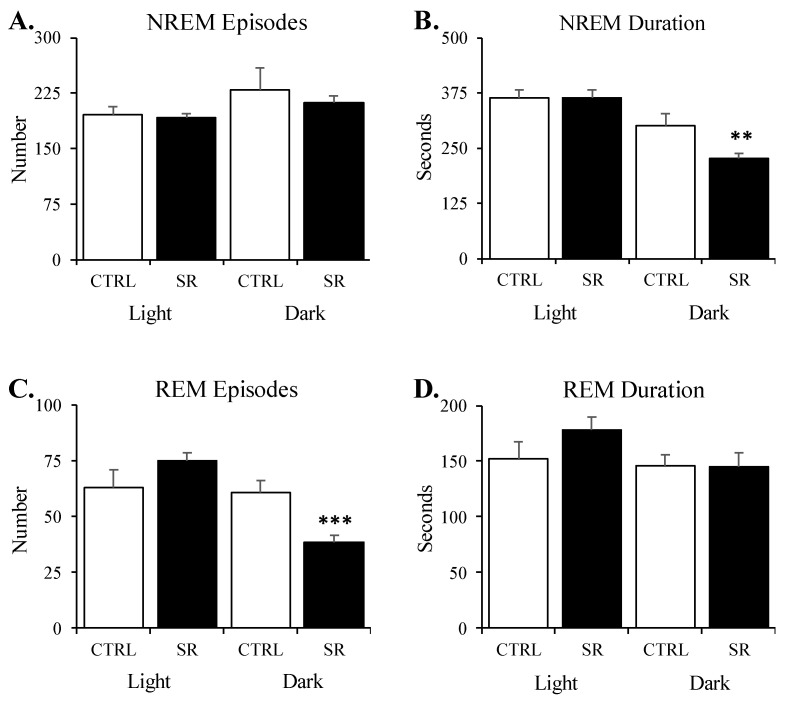
**Selected sleep parameters for NREM and REM in control (CTRL) and irradiated (SR) rats during the 8 h light period and 12 h dark period**. (**A**) nonrapid eye movement (NREM) episode number; (**B**) NREM episode duration; (**C**) rapid eye movement (REM) episode number; (**D**) REM episode duration. **, *p* < 0.01; ***, *p* < 0.001 for comparisons between CTRL (*n* = 15) and SR (*n* = 15). Data are presented as mean ± SEM.

During the light period, the amounts of TST, NREM, and REM did not significantly differ between the groups (Figure 1A–C). There were also no significant differences between the number and durations of NREM and REM episodes (Figure 2A–D) or in REM% (Figure 1D).

In the dark period, the amounts of TST (t(28) = 2.57, *p* < 0.05), NREM (t(28) = 2.45, *p* < 0.05), and REM (t(28) = 3.79, *p* < 0.001) were significantly reduced in the SR rats (Figure 1A–C). The differences in the number of NREM episodes (*p* = 0.18) did not reach significance (Figure 2A), but there was a significant reduction in NREM episode duration (Figure 2B) for SR rats compared to sham rats (t(28) = 3.20, *p* < 0.01). The SR-related reductions were found in the number of REM episodes (t(28) = 3.76, *p* < 0.001; Figure 2C), whereas REM episode durations did not significantly differ (Figure 2D).

### 3.2. EEG Spectra

**Figure 3:**
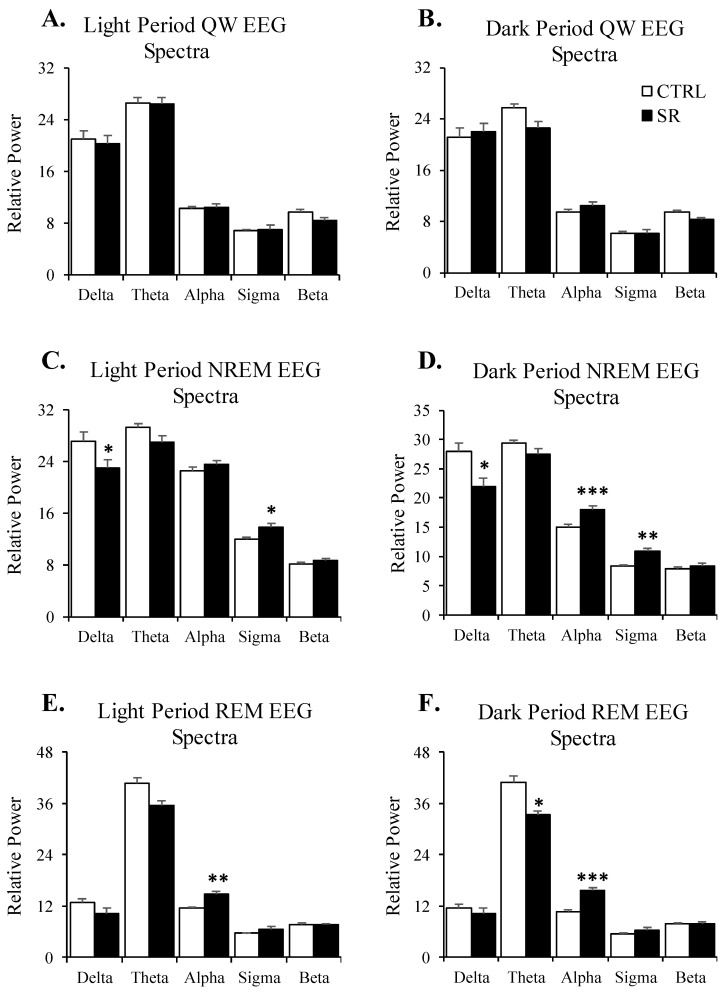
**EEG spectra plotted for quiet waking (QW), nonrapid eye movement sleep (NREM), and rapid eye movement sleep (REM) in the control (CTRL; *n* = 15) and irradiated (SR; *n* = 15) rats during the 8 h light period and 12 h dark period.** (**A**) QW EEG spectra during the light period; (**B**) QW EEG spectra during the dark period; (**C**) NREM EEG spectra during the light period; (**D**) NREM EEG spectra during the dark period; (**E**) REM EEG spectra during the light period; (**F**) REM EEG spectra during the dark period. * *p* < 0.05; **, *p* < 0.01, *** *p* < 0.001 for comparisons between CTRL (*n* = 15) and SR (*n* = 15) within spectral bands. Data are presented as mean ± SEM.

No significant differences between the CTRL and SR rats were found in the analysis of the EEG spectra for QW during either the light (Figure 3A) or dark (Figure 3B) periods.

The light period EEG in NREM in the SR rats (Figure 3C) showed significant decreases in delta (t(28) = 2.05, *p* < 0.05) and significant increases in the sigma band (t(28) = 2.68, *p* < 0.02) compared to CTRL rats. For the dark period NREM (Figure 3D), the SR rats showed a decreased EEG delta power (t(28) = 2.66, *p* < 0.02) and an increased EEG alpha (t(28) = 3.95, *p* < 0.001) and sigma (t(28) = 3.88, *p* < 0.001) power compared to the CTRL rats.

For the light period EEG in REM (Figure 3E), increased power in the alpha (t(28) = 2.98, *p* < 0.01) band in the SR rats was found when compared to CTRL rats. For the dark period REM (Figure 3F), the SR rats showed decreases in theta (t(28) = 2.24, *p* < 0.04) and increases in EEG alpha (t(28) = 3.85, *p* < 0.001) power compared to the CTRL rats. No other apparent changes in the EEG spectral powers were observed for NREM or REM.

### 3.3. Activity

**Figure 4:**
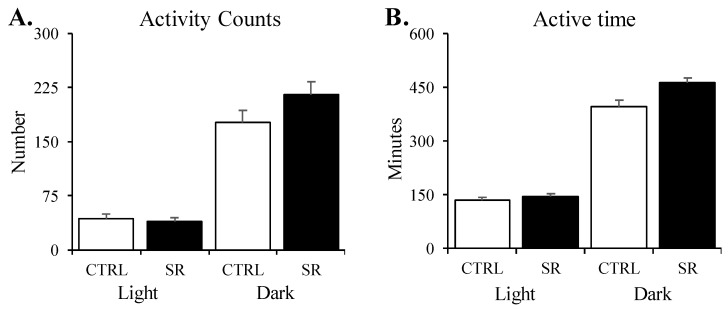
**Total activity counts (A) and active time (B) in the control (CTRL) and irradiated (SR) rats during the 8 h light period and 12 h dark period**. There were no significant differences between the groups for either period. CTRL (*n* = 15) and SR (*n* = 15). Data are presented as mean ± SEM.

No significant differences were found between the CTRL and SR rats for activity, considered as either total activity counts (Figure 4A) or active time (Figure 4B) regarding the 8 h light or 12 h dark periods.

### 3.4. Temperature

**Figure 5:**
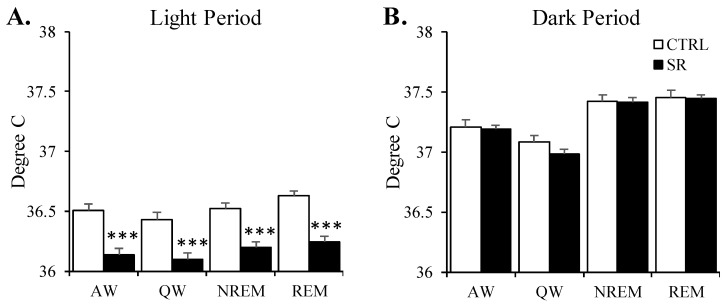
**Core body temperature (CBT) plotted for active waking (AW), quiet waking (QW), nonrapid eye movement sleep (NREM), and rapid eye movement sleep (REM) in the control (CTRL) and irradiated (SR) rats during the 8 h light period and 12 h dark period.** (**A**) CBT in the light period; (**B**) CBT in the dark period. ***, *p* < 0.001 for comparisons between CTRL (*n* = 15) and SR (*n* = 15). Data are presented as mean ± SEM.

CBT during the light period (Figure 5A) was significantly reduced in the SR rats in all analyses: QW (t(28) = 3.95, *p* < 0.001); AW (t(28) = 5.01, *p* < 0.0001); NREM (t(28) = 4.46, *p* < 0.001), and REM (t(28) = 3.83, *p* = 0.001) when compared to CTRL rats. The reductions in all states were approximately 0.5 °C (AW: −0.34 °C; QW: −0.37 °C; NREM: −0.32 °C; REM: −0.39 °C). In the dark period, there were no significant differences in CBT across the groups (Figure 5B).

### 3.5. Hourly Alterations in Activity, Sleep, and Temperature

**Figure 6:**
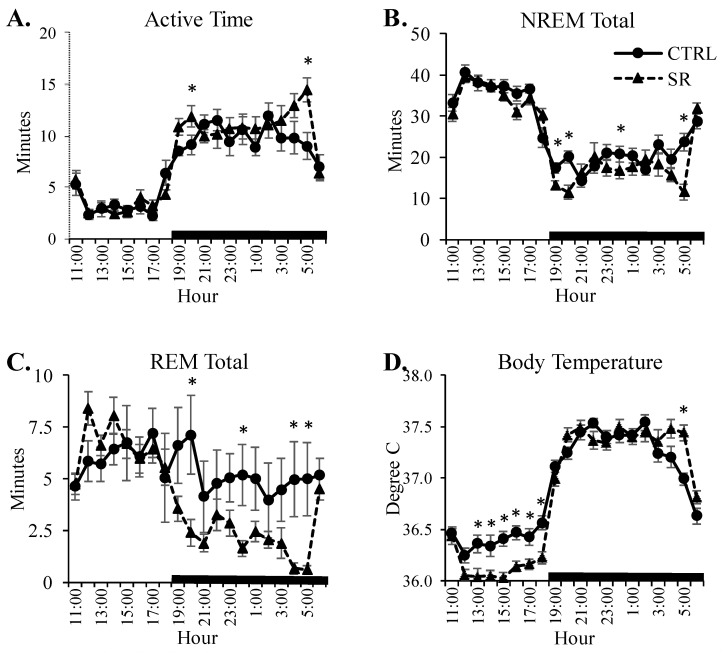
**Hourly plots of select parameters in the control (CTRL) and irradiated (SR) rats.** (**A**) active time; (**B**) nonrapid eye movement sleep (NREM); (**C**) rapid eye movement sleep (REM), and (**D**) core body temperature. *, *p* < 0.05 for comparisons of CTRL (*n* = 15) and SR (*n* = 15). Dark bar indicates dark period. Data are presented as mean ± SEM.

Active time showed a significant Group × time interaction: F(19,532) = 1.939, *p* = 0.048. However, there were only two time points during the dark period in which activity in the SR rats increased above that of the CTRL rats (Figure 6A).

There were significant Group × time interactions for the hourly total NREM: F(19,532) = 2.831, *p* = 0.03, and for the hourly total REM: F(19,532) = 3.941, *p =* 0.015. Significant differences between the CTRL and SR rats for both NREM (Figure 6B) and REM (Figure 6C) were observed only during the dark period.

The ANOVA for CBT revealed a significant Group × time interaction: F(19,532) = 7.887, *p* < 0.001. Significant reductions in CBT were found during 6 h of the light period and a significant increase during 1 h of the dark period (Figure 6D).

## 4. Discussion

The current results demonstrate that SR approximating the amount that astronauts will receive on the Mars mission can significantly alter baseline sleep parameters, EEG spectra, and CBT. The overall alterations in sleep amounts, episode numbers and durations, and activity, particularly in the light period, are relatively small and suggest that SR at this dosage may not alter overall sleep drive or arousal. More prominent are the significant alterations in the EEG spectra during both the light period and dark periods that suggest that sleep state organization and efficiency may be reduced, with a potential impact on overall arousal and performance. A reduction in CBT during only the inactive period when sleep is greatest may also have interactions with sleep.

The baseline sleep recordings in this study were conducted in relatively consistent environmental conditions with minimal external disruptions, both before and during the recording periods. However, studies have found that, for multiple reasons (e.g., noise, physical discomfort, altered light-dark cycles, varying work shifts, hypoxia, hypercapnia, and psychological factors), astronauts in space sleep less than they normally do on Earth [7,8,9,10]. Sleep quality and structure can also be altered. Some changes in sleep that have been reported include altered latency to REM, shorter REM episode duration, and the redistribution of NREM between the first and the second sleep cycles [45]. There is also a 27–50% reduction in REM and NREM time in space compared to that on Earth [46,47,48], and astronauts living on the international space station (ISS) exhibit increased sleep pressure (greater theta activity in the EEG and more local sleep events during waking) [49]. While there are variations across studies, the evidence indicates that sleep in space will be subject to disruptions associated with a number of environmental factors. Thus, the alterations in sleep regulation produced by SR will occur in a stressful environment that already produces significant reductions in sleep, suggesting the possibility that SR could exacerbate the effects of stress.

Essentially, all stressors have the potential to impact sleep [50,51], and poor sleep quality can reduce the ability to cope with stressful experiences [52,53,54,55], at least partially through negative effects on cognition and emotion [52]. Sleep deprivation in healthy adults can result in both an elevation in resting cortisol levels and greater cortisol release in response to a stressor, indicative of elevated HPA axis activity [56]. We have also found that even brief periods of sleep fragmentation after SR (10 cGy Si) in rats can increase sleep disruptions and compromise the ability of individual animals to show recovery sleep responses [22].

Sleep is also intimately linked to the immune system [57,58,59], and the reciprocal influences of sleep and the immune system have led to the suggestion that sleep is a component of the acute phase response to infection [57] and that it functions in host defense [59]. Post-stress sleep may also be important for mediating the effects of stress on the immune system, as insufficient sleep can promote pro-inflammatory cytokines [60], and disrupted sleep both before and after significant stress is implicated in stress-related pathology [52,55]. Experimentally fragmented sleep, even without alterations to total sleep time, can impact the immune system [61], and both sleep disruption [62,63] and REM restriction [64] disrupt the function of the blood-brain barrier by altering its neuroimmune regulation [62,65].

This study used middle-aged animals in an attempt to match the likely age of astronauts who will serve on the Mars missions. In humans, sleep disturbances increase with aging [66,67], and most age-dependent changes in sleep architecture occur before the age of 60 years [68], although some parameters can continue to deteriorate in later life (reviewed in [69]). Studies in middle-aged rats also indicate alterations in sleep quality, but little evidence of age-related change in 24 h total sleep at midlife [70,71,72]. Comparisons of 3-month and 1-year-old rats found no age-related changes in total sleep, NREM or REM sleep, or wake time after sleep onset, but did find significant age-related reductions in high-voltage NREM sleep, the mean length of sleep bouts, and REM-onset duration, indicating that changes to sleep in animals can be evident by midlife [72]. Human studies of REM sleep have tended to show an age-related decline in absolute amounts (reviewed in [69]), and some studies in rats have also indicated modest [70,73] declines in REM. Thus, the effects of SR need to be considered in the context of sleep that, even in healthy humans, is already altered with age.

We examined the relative power of five spectral bands: delta, 0.5–4 Hz; theta, 4–8 Hz; alpha, 8–12 Hz; sigma, 12–16 Hz; beta, 16–24 Hz. The SR rats showed significant decreases in NREM delta power (light and dark periods) and significant increases in sigma (light and dark periods), and increases in alpha (dark period) power. NREM-associated EEG delta activity is considered a measure of potential changes in sleep intensity [74,75,76]. Additionally, the SR rats also showed increased alpha band power during light and dark period REM. While the significance of these increases in the higher frequency bands is not fully understood, studies have suggested that increased spectral power in high-frequency bands in an EEG may be a hyperarousal marker of insomnia [77,78]. Lower NREM delta and greater alpha, sigma, and beta EEG activity have also been reported in patients with subjective, but not objective, insomnia [79]. Thus, reductions in delta and increases in the higher frequency bands suggest altered and potentially less restorative sleep after SR exposure.

The SR rats also showed significant reductions in dark period REM theta power and a nonsignificant trend (*p* < 0.09) towards decreased light period REM theta power REM. In a small study, we also found that exposure to 20 cGy 1 GeV/n ^56^Fe irradiation led to a marked reduction in peak magnitude theta wave activity during REM ([80]). Thus, SR has the potential to significantly alter theta activity in REM. This might be significant, as the general consensus is that the large-scale theta oscillation detected in the EEG, as recorded and analyzed in our study, is primarily produced in the hippocampus [81] and that changes in power amplitude arise from macroscopic changes in synchronization within local neural ensembles [82]. Accordingly, the changes in theta activity that we observed likely reflect neural activity in the hippocampus [83]. Recently, a functional role of theta oscillations has been hypothesized as a means to enable network-level co-operation for the collective actions of single neuron computations underlying cognitive functions [84,85], including memory consolidation [86] and fear extinction [87]. Thus, the reductions we observed in baseline REM theta may have consequences for information processing at the network level.

In the SR rats, we observed significant decreases in light period CBT of greater than 0.3 °C during all sleeping and waking states, whereas, during the dark period, there was only a single 1 h period in which both increased CBT and increased activity were observed in the SR rats. Endogenous rhythms in body temperature in rats are at their lowest during the light phase when animals are generally sleeping, and these increase during darkness when the animals are active. All of the animals were held in the same conditions and rooms, suggesting a dysfunction in the animal’s body temperature regulation after SR. In mammals, CBT is controlled within a narrow range by the preoptic nucleus of the hypothalamus, and staying within this range is important for maintaining physical [88] and cognitive [89] performance. The circadian rhythms of sleep and temperature also are normally coupled with reductions in temperature occurring prior to the onset of NREM [90]. Body temperature is also associated with exercise; however, we saw no difference in light-period activity between the CTRL and SR animals. The integration of arousal state and thermoregulation is controlled by the preoptic/anterior hypothalamus [91,92], suggesting areas to examine for potential mechanisms as to the reduced light period temperature.

Interestingly, in space, CBT rises higher and faster during physical exercise and baseline CBT becomes elevated during long-duration spaceflight [42]. These increases have been suggested to be related to persistent low-grade pro-inflammatory responses to weightlessness, strenuous exercise protocols, radiation, psychological stress-induced hyperthermia, or some combination of factors and have implications for astronaut health, well-being, and support, including energy, nutrient, and fluid requirements, as well as physical and cognitive performance [42]. The current results suggest that SR-induced alterations to temperature control have the potential to interact with the other effects that spaceflight may have on CBT. The effects on temperature control may be widespread, as a proteomic analysis of the prefrontal cortex of rats exposed to SR (15 cGy of Si) identified several pathways that significantly differed from the CTRL rats, including thermogenesis [93].

Our study demonstrates that SR can alter sleep and temperature regulation under undisturbed conditions in ways that may have consequences for astronaut health and performance, especially in the stressful environment that will be encountered on long space missions. However, additional work is needed to fully understand these SR-induced alterations. For example, our data are suggestive of circadian differences in sleep, EEG spectra, and CBT, which we did not assess in this study. In the future, it would be useful to assess any potential day-to-day variability as well as any potential alterations to the circadian regulation of sleep and CBT. We also did not report on whether there are potential sex differences with respect to the effects of SR on sleep. Several studies report that female rodents are more resilient to the long-term effects of SR exposure regarding novel object recognition [31,94,95,96,97] and pattern separation [94], whereas others report impaired novel object recognition [98]. In general, the resiliency of female rodents to SR appears to be highly context specific. For example, while pattern separation is differentially preserved in female compared to male mice [23,94], performance in a striatal-dependent stimulus-response rule-based habit-learning task is impaired in female mice [94]. In addition, GCRSim-exposed female rats had increased levels of anterograde interference within the associative recognition memory and interference touchscreen (ARMIT) task [99]. Given the role of sleep in cognition, determining sex differences regarding how it may be altered by SR could provide clues as to the reasons for sex differences on performance, and why results may vary across studies. We are currently conducting parallel studies in females and will provide comparisons of baseline sleep in males and females in the future.

Additionally, we have found differences in stress-resilient and vulnerable phenotypes based on sleep responses to stress [100,101,102,103] regarding the effects of sleep fragmentation after exposure to 10 cGy Si SR. After SR exposure, vulnerable rats showed increased sleep disruption during SF, whereas resilient rats showed similar responses to SF both before and after SR. Resilient rats also showed modest (and rapidly recovered from) NREM disruption and essentially no REM alteration, whereas vulnerable rats showed delayed recovery for both NREM and REM. Thus, SR may differentially impact the ability of individual astronauts to cope with the stressors they encounter. In going forward, more in-depth examinations of SR-induced changes in sleep regulation, EEG spectra, and related systems and their relevance regarding performance are needed. Additionally, there is a need for an examination of how SR-induced alterations to sleep and CBT interact with the stressors (e.g., social isolation and microgravity) that astronauts will experience on deep space missions, as well as mission-related cognitive and emotional challenges. Because sleep plays an important role in performance and health, understanding how it is impacted by SR and spaceflight stressors may provide avenues to mitigate some of their deleterious effects and support mission success.

In summary, our results demonstrate that, compared to the CTRLs, SR produces significant reductions in the amounts of dark period total sleep time, total NREM, and total REM sleep and significant decreases in light and dark period NREM delta and dark period REM theta, as well as increases in alpha and sigma in NREM and REM during either light or dark periods. The SR animals also showed modest increases in some measures of activity and a modest reduction in CBT during waking and sleeping in the light period. These data demonstrate that SR alone can produce alterations to sleep and temperature control that could have consequences for astronauts and their ability to meet mission demands. Critically, SR-induced alterations to sleep system integrity could make astronauts less able to withstand the adverse effects of the stressors they will encounter on deep space missions.

## Data Availability

Experimental data available upon request.

## Supplementary Materials

The following supporting information can be downloaded at: https://www.mdpi.com/article/10.3390/life13041002/s1, Table S1: Select sleep parameters comparing non-shipped (Control) and shipped (Sham) rats; Figure S1: Number of Control and Sham animals that reached criterion performance during balance beam testing. Figure S2: Success rates for Control and Sham groups after an animal had successfully met Criterion performance on the balance beam.
