# Supplementary material for: Sleep and Core Body Temperature Alterations Induced by Space Radiation in Rats
Source: Life (Basel). 2023 Apr 13;13(4):1002. doi: 10.3390/life13041002 (PMC10144689; doi:10.3390/life13041002)

Table 1. Select sleep parameters comparing non-shipped (Control) and shipped (Sham) rats. Data are mean ± SEM presented as 20 h totals. There were no significant differences across groups.

Parameter	Control	Sham
**Total Sleep Time**	543.4 ± 7.6	559.1 ± 18.9
**Total NREM sleep**	487.2 ± 10.3	459.9 ± 12.5
**Total REM sleep**	78.1 ± 7.0	80.4 ± 15.7

Figure 1. Number of Control and Sham animals that reached criterion performance during balance beam testing. Criterion for successful learning was determined by an individual animal reaching the goal box starting from the farthest position.

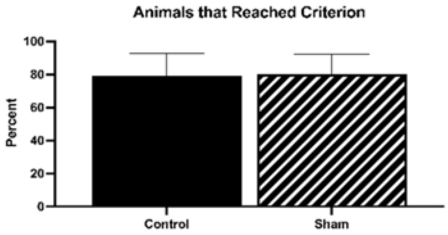

Figure 2. Success rates for Control and Sham groups after an animal had successfully met Criterion performance on the balance beam. Percent success was calculated for the animals within a group that reached criterion based on the number of successful trails from the farthest position over the total number of trials attempted at that position (%Success = # of Successful Trials at Criterion/Total # of Trials at Criterion x 100). The difference between groups was not significant (p=.07).

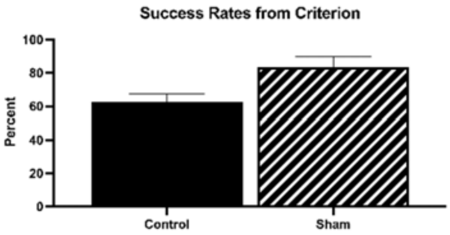

Supplement: Supplementary file 1 [file life-13-01002-s001.zip › life-2218889-supplementary.pdf]
